# Supplementary material for: Zinc Finger Protein 90 Knockdown Promotes Cisplatin Sensitivity via Nrf2/HO-1 Pathway in Ovarian Cancer Cell
Source: Cancers (Basel). 2023 Mar 3;15(5):1586. doi: 10.3390/cancers15051586 (PMC10000492; doi:10.3390/cancers15051586)
Supplement: Supplementary file 1 [file cancers-15-01586-s001.zip › cancers-2238850-supplementary.pdf]

Supplementary Materials: Zinc Finger Protein 90 Knockdown Promotes Cisplatin Sensitivity via Nrf2/HO-1 Pathway in Ovarian Cancer Cell

Ching-Hu Wu, Chien-Wei Feng, Chiu-Lin Wang, Zhi-Hong Wen, Cheng-Yu Long and Feng-Hsiang Tang

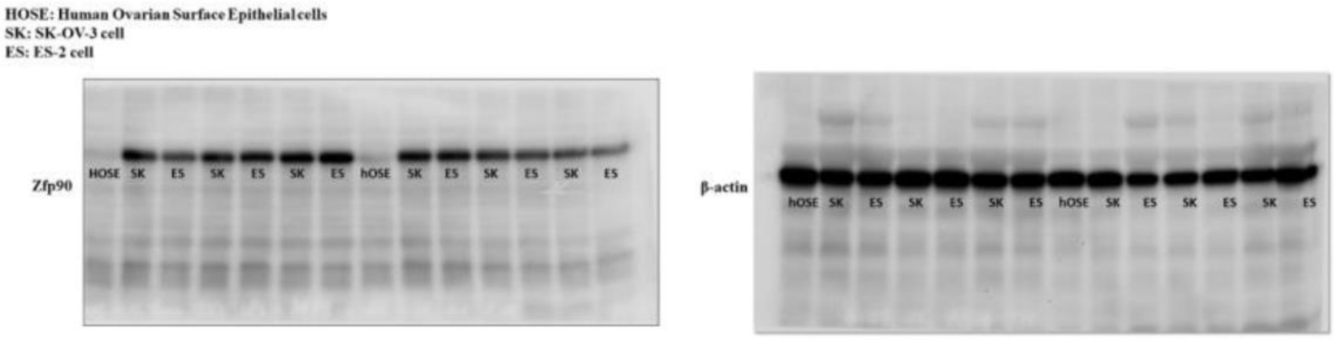

Figure S1. The uncropped western membrane of Figure 1G (Zfp90 and β-actin in human ovarian surface epithelial cells (HOSE), ES-2 cell, SK-OV-3 cell).

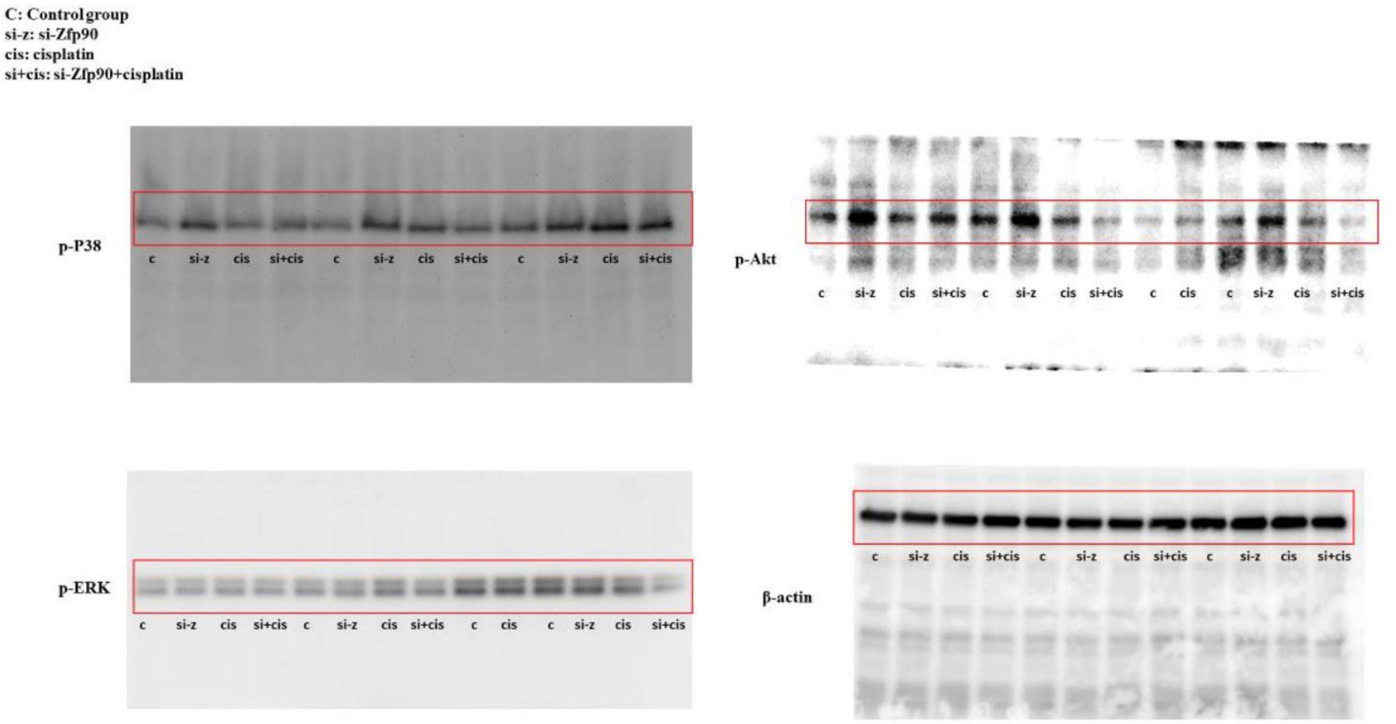

Figure S2. The uncropped western membrane of Figure 2A of p-P38, P38, p-ERK, ERK, p-Akt and Akt in SK-OV-3 cell.

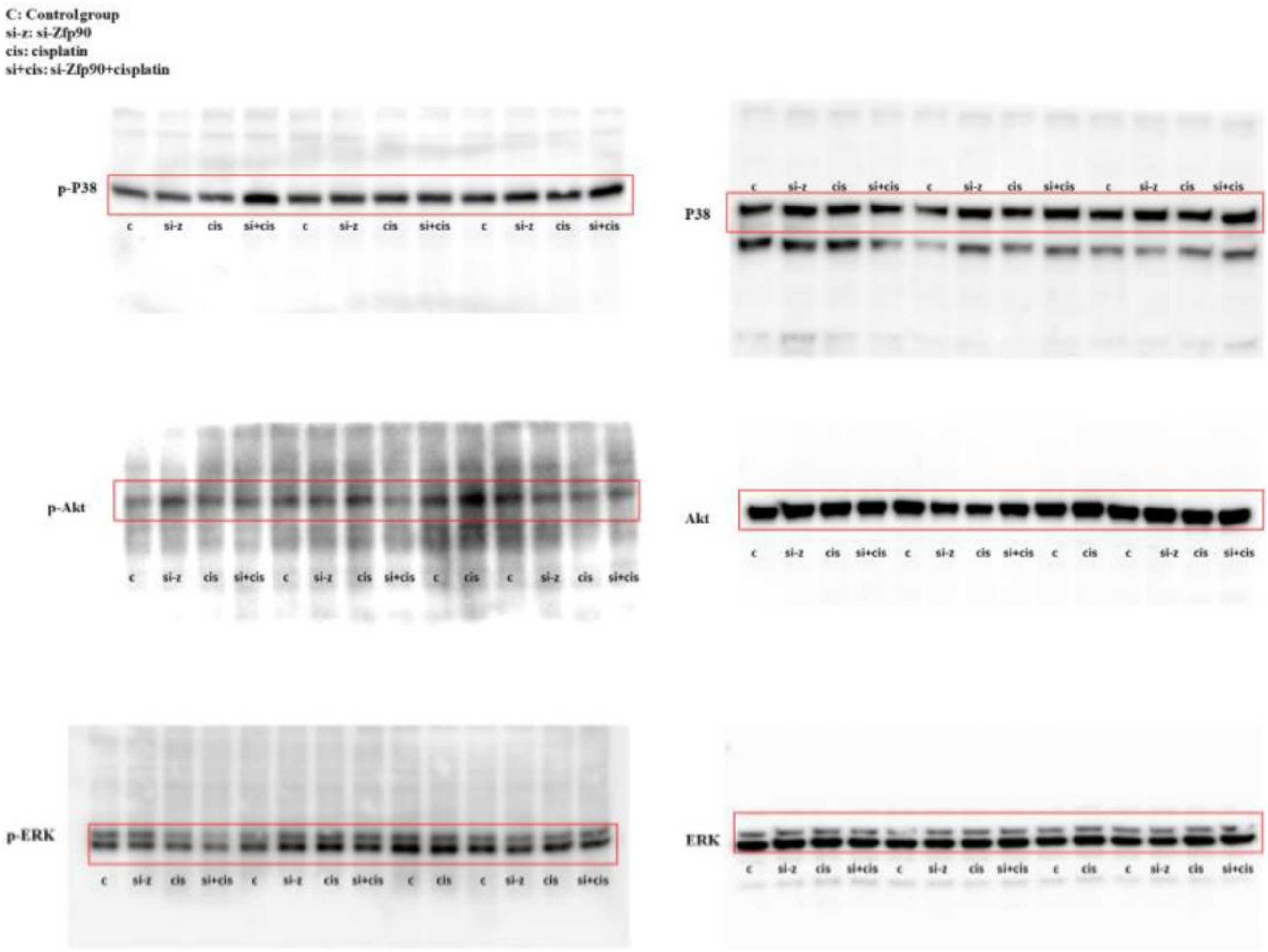

Figure S3. The uncropped western membrane of Figure 2E of p-P38, P38, p-ERK, ERK, p-Akt and Akt in ES-2 cell).

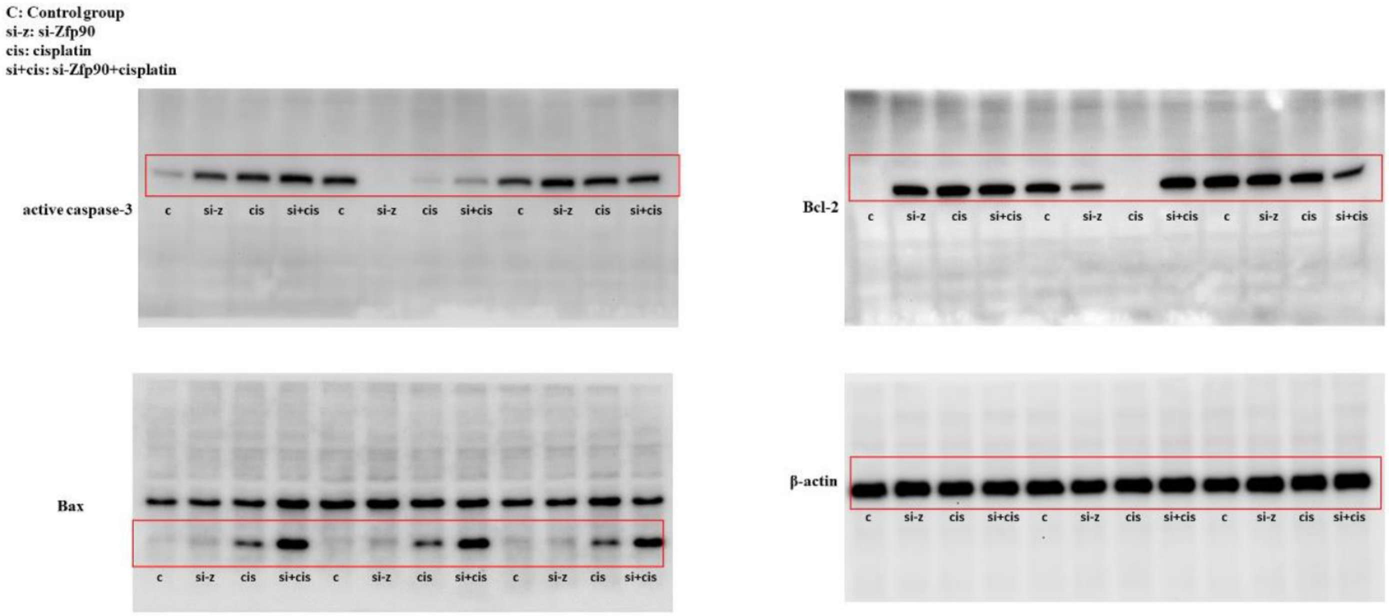

Figure S4. The uncropped western membrane of Figure 3A of active caspase-3, Bcl-2, Bax and β-actin in SK-OV-3 cell.

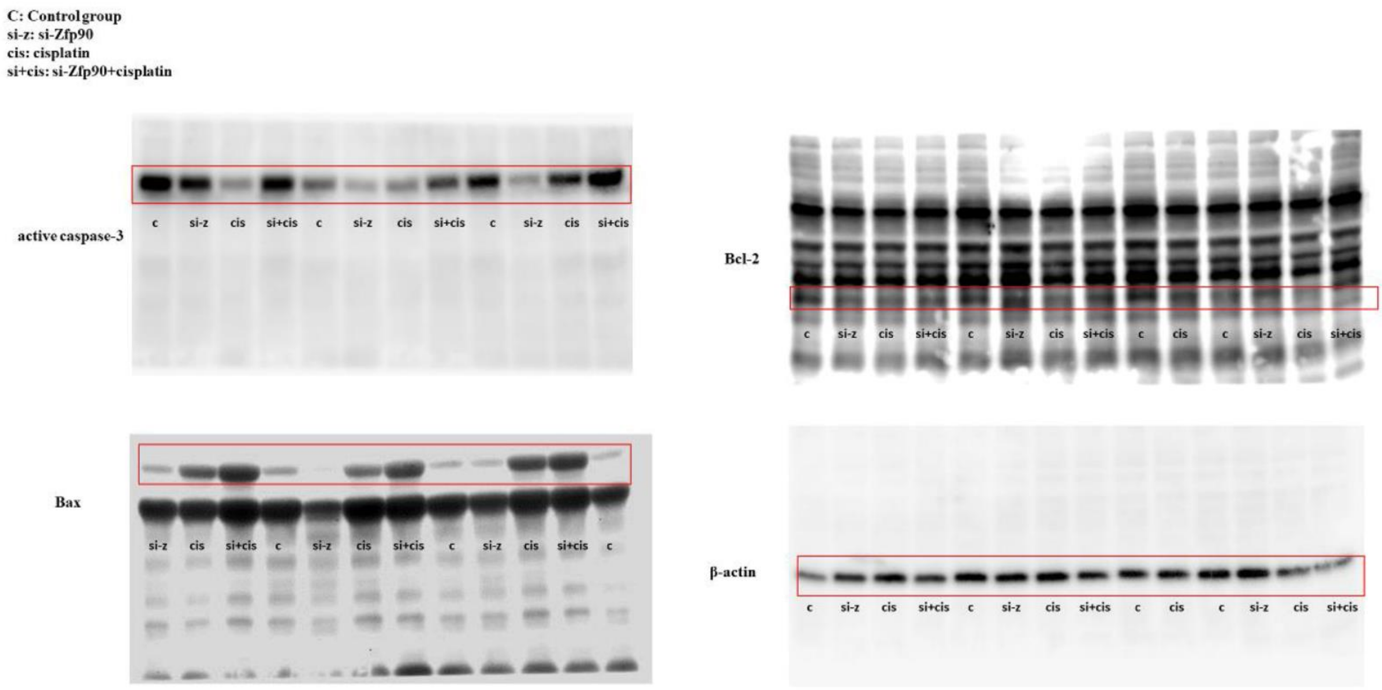

Figure S5. The uncropped western membrane of Figure 3E of active caspase-3, Bcl-2, Bax and β-actin in ES-2 cell.

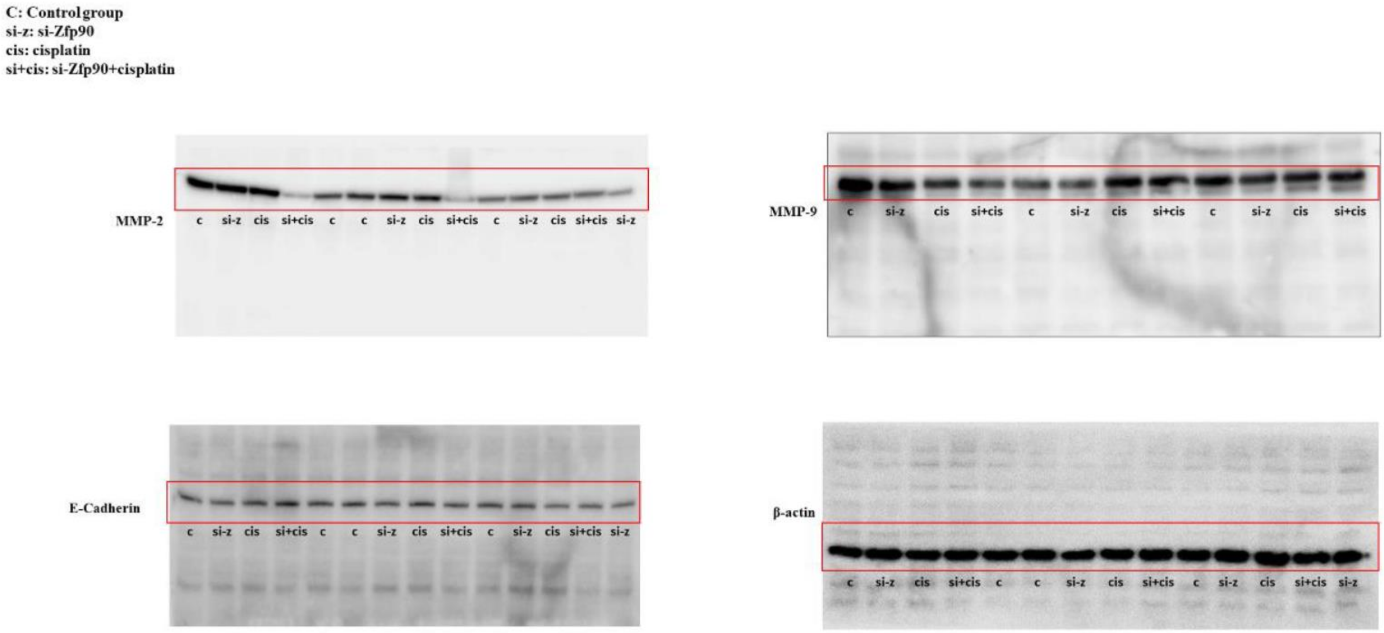

Figure S6. The uncropped western membrane of Figure 4D of MMP-9, MMP-2, E-cadherin and β-actin in SK-OV-3 cell.

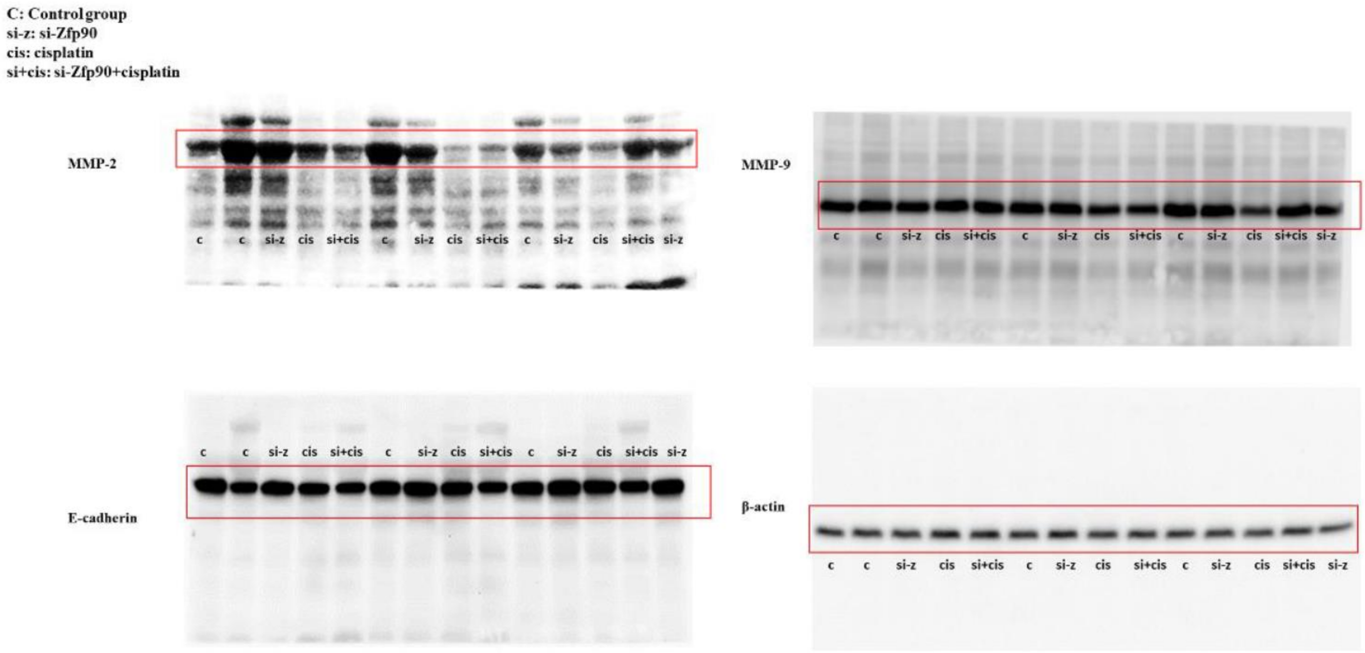

Figure S7. The uncropped western membrane of Figure 4K of MMP-9, MMP-2, E-cadherin and β-actin in ES-2 cell.

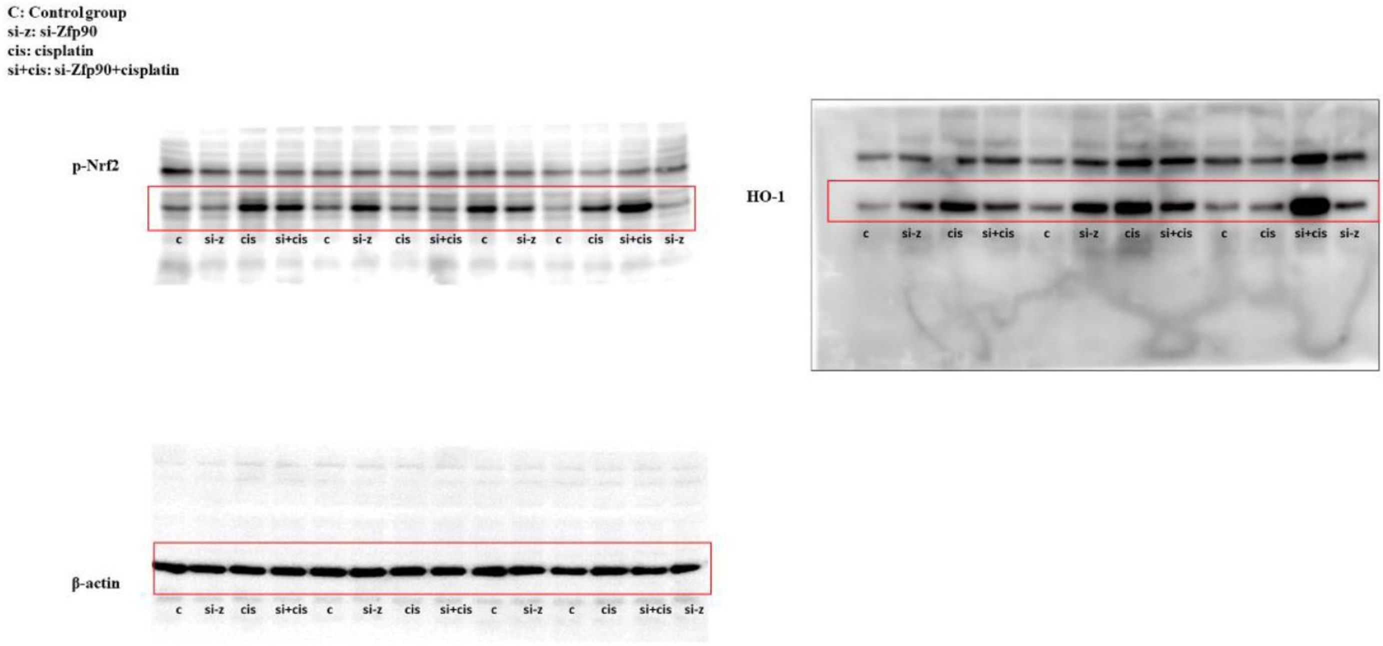

Figure S8. The uncropped western membrane of Figure 5A of p-Nrf2, HO-1 and β-actin in SK-OV-3 cell.

C: Control group
si-z: si-Zfp90
cis: cisplatin
si+cis: si-Zfp90+cisplatin

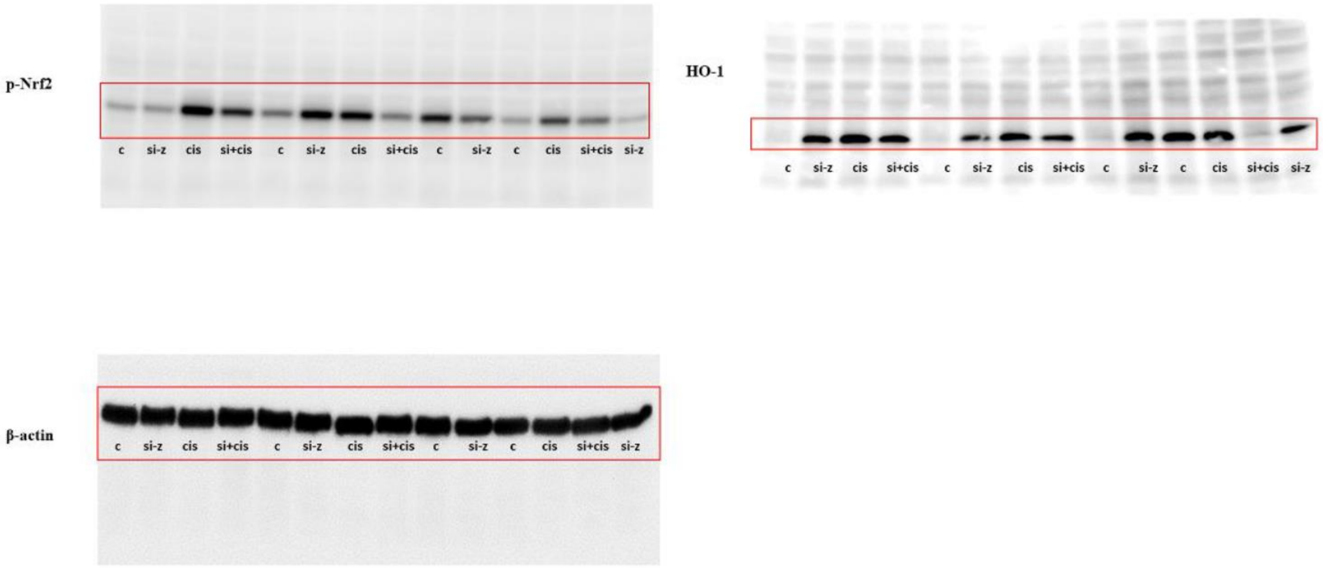

Figure S9. The uncropped western membrane of Figure 5E of p-Nrf2, HO-1 and β-actin in ES-2 cell.
